# Genetic analysis of a SARS-CoV-2 Omicron variant from a Chinese traveller returning from overseas

**DOI:** 10.1080/22221751.2022.2025747

**Published:** 2022-02-04

**Authors:** Hongling Jia, Hui Wang, Lan Cao, Zhisheng Lai, Zichun Cheng, Qingpei Chen, Tongzheng Liu, Xiangyi Liu, Yanting Wen, Conghui Xu, Weizhi Lu, Guang Yang, Deqian Zhou, Biao Di, Feng Gao, Zhicong Yang

**Affiliations:** a Guangzhou Center for Disease Control and Prevention, Guangzhou, People’s Republic of China; b Department of Medical Biochemistry and Molecular Biology, School of Medicine, Jinan University, Guangzhou, People’s Republic of China; c Institute of Molecular and Medical Virology, School of Medicine, Jinan University, Guangzhou, People’s Republic of China; d Yuexiu District Center for Disease Control and Prevention, Guangzhou, People’s Republic of China; e Clinical Data Center, Guangdong Provincial People’s Hospital, Guangdong Academy of Medical Sciences, Guangzhou, People’s Republic of China; f College of Pharmacy, Jinan University, Guangzhou, People’s Republic of China; g Department of Pathogen Biology, School of Medicine, Jinan University, Guangzhou, People’s Republic of China

**Keywords:** SARS-CoV-2, Omicron variant, mutation, infection, COVID-19

## Abstract

Since the SARS-CoV-2 Omicron variant was first reported from South Africa, it has rapidly spread in over 100 countries. Only two cases infected by the Omicron variant were recently identified in China. The one case in Guangzhou has a relatively long incubation time and mild symptoms. Analysis of the complete viral genome sequence shows three missing Omicron unique mutations and one additional mutation in the newly characterized genome. These unique mutations may be related to the clinical presentation in this case.

A new variant (B.1.1.529) of severe acute respiratory syndrome coronavirus 2 (SARS-CoV-2) was first detected in South Africa [1]. Since it rapidly outpaced other SARS-CoV-2 strains in South Africa and quickly spread to other countries [2], the World Health Organization (WHO) soon designated it as a variant of concern (VOC) and later named it Omicron [3]. It contains many more mutations than any previous VOCs; a total of 47 mutations in the whole genome, including 26 mutations in the spike (S) gene, and 13 mutations alone in the receptor-binding domain (RBD) in the S gene [4]. Moreover, five new mutations were found at both ACE2 and antibody binding sites, suggesting an increased immune escape capability [5]. Some studies indicate that the Omicron variant might have increased transmissibility, enhanced viral binding affinity and elevated antibody escape [1,2,6–8]. The *in vitro* neutralization assay shows that the mean neutralization titres by sera from convalescent COVID-19 patients are significantly reduced against the Omicron variant than other variants [7,8].

While China was initially spared with the Omicron variant, one 67-year-old male (Case 1) was found infected with the SARS-CoV-2 Omicron variant in Guangzhou, Guangdong, China on December 13, 2021, the same day when the infection case of the Omicron variant was reported in Tianjin, China [9]. He received the SARS-CoV-2 Pfizer-BioNTech COVID-19 vaccine on April 7 and May 8, 2021, in Phoenix, Arizona, U.S.A. He left Phoenix for Victoria, British Columbia, Canada on September 11, and stayed there until November 25 when he left for China. He denied any close contacts with COVID-19 patients or people with symptoms. He arrived at Shanghai Pudong International Airport on November 27 and was immediately transported to a local hotel, where he was quarantined for 14 days in isolation. Oropharyngeal swabs were collected on day 1, 4, 7, and 14 (December 10) of the quarantine period and the PCR results were all negative for COVID-19 nucleic acid by qPCR. He left Shanghai for Guangzhou by airplane on December 11. An oropharyngeal swab was collected for the routine PCR test in the morning on December 12 after he arrived in Guangzhou. In the evening of December 12, he developed mild symptoms like fever, xerostomia, expectoration, and cough, with a body temperature of 37.7°C. The PCR test was positive of SARS-CoV-2 in the morning on December 13. Tests were repeated three times and they were all positive. He was immediately transferred to the local hospital specialized in infectious diseases. Serological tests showed that he was negative for SARS-CoV-2-specific IgM but positive for IgG. Lastly, Clinical examination showed increased and thickened bronchial vascular bundles in both lungs and a ground glass-like appearance in the posterior segment of the right upper lobe. He was diagnosed as a mild case of COVID-19.

One 70-year-old female (Case 2) and her 41-year-old daughter (Case 3), who both had been vaccinated with the full course of whole inactivated vaccine and was living in the same building with Case 1 in Guangzhou after his quarantine, were transferred to the quarantine station on December 13 after the Case 1 was diagnosed positive for SARS-CoV-2 infection. They lived in the connected two rooms in the station. They were both negative for SARS-CoV-2 infection by the nucleic acid test for next consecutive days. However, Case 2 and Case 3 were found positive for SARS-CoV-2 infection on December 16 and 17, respectively. Both were immediately transferred to the local hospital specialized in infectious diseases and diagnosed as a mild case of COVID-19.

Nasal swabs collected from Cases 1, 2, and 3 were used to generate the complete SARS-CoV-2 genome sequence using Nanopore GridION and validated by using Illumina HiSeq. The sequencing data were assembled from each sample and analysed using the Geneious package. Phylogenetic analysis of the whole genome sequence showed that three sequences were cluster together and closely related to one of the Omicron lineage BA.1 (Figure 1A). The genetic distance between them and BA.1 sequences is 0.01%. It is much less divergent than those between them and BA.2 sequences (0.11%) or B.1.1.529 sequences (0.07%). The Case 1 and Case 2 sequences were identical, while the Case 3 sequence different by one mutation that resulted in a P3395H mutation in ORF1a (Figure 1B). Two Omicron signature mutations (K417N in the S gene and D3G in the M gene) were not detected in all three sequences, while the P3395H mutation in the ORF1a gene was not found in Cases 1 and 2, but present in Case 3 (Figure 1B). The T333M mutation in the ORF1a gene was detected in all three sequences, but not found in currently known Omicron variant sequences. This unique new set of sequences explains the relatively long branch in the phylogenetic tree (Figure 1A), suggesting that all three cases are infected by a particular Omicron variant.

**Figure 1.**
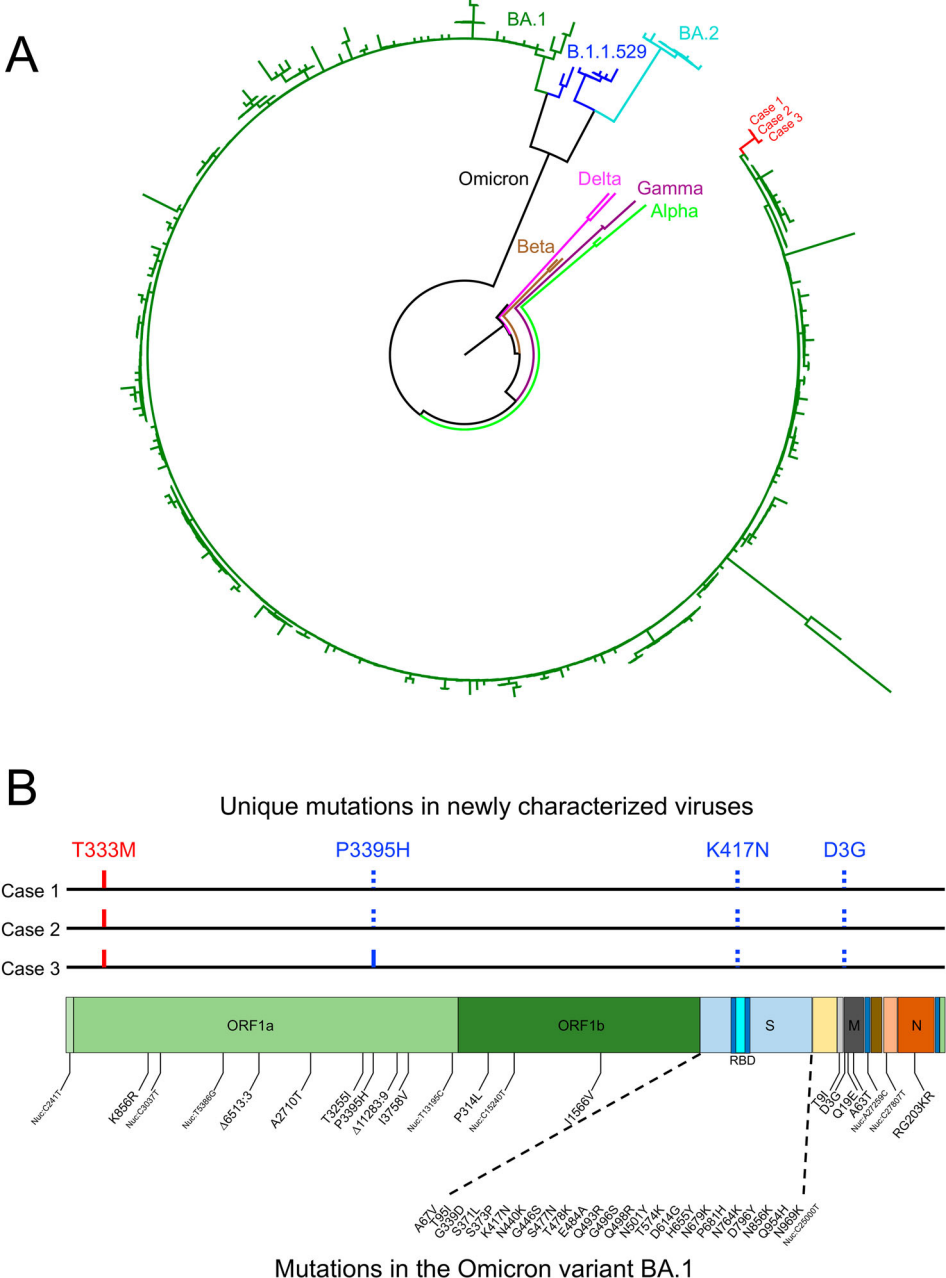
Sequence analysis of the newly characterized SARS-CoV-2 Omicron variant sequences. (A) Phylogenetic tree was constructed by the maximum likelihood method using the GTR model for the complete genome sequences from Cases 1, 2, and 3. Three different clades of the Omicron variant as well as reference sequences from Alpha, Beta, Gamma, and Delta. The three new sequences are shown in red. (B) Locations of unique mutations in the SARS-CoV-2 genome. The mutations that are in Omicron BA.1 but not in the new sequences (dotted blue ticks) and the mutations that not found in Omicron BA.1 (red ticks) in the new sequences are indicated. The P3395H mutation that was found in Case 3 but not in Cases 1 and 2 is indicated by the solid blue tick. The signature mutations in the Omicron BA.1 are shown below the genome structure.

The whole-genome sequences from Cases 1 and 2 are identical, while Case 3 genome only one mutation that different from Case 1 and 2 genomes. Since Case 3 was found positive for SARS-CoV-2 infection only 1 day after Case 2 was confirmed, both Cases 2 and 3 must have been infected by the same virus from Case 1 through close contact. Considering only two people being infected by this particular Omicron variant in China and the relative short time window during which Cases 2 and 3 were infected, the transmissibility of this particular Omicron variant in China might be underestimated and long-term monitoring is required.

Repeated negative results of PCR amplification of SARS-CoV-2 genes and no detection of SARS-CoV-2-specific IgM suggest that Case 1 was infected recently. It is also possible that the initial infection was severely suppressed by the immunity elicited by the vaccine so the virus only replicated at low levels initially for an extended period until it successfully established a clinical infection after more than 2 weeks of the incubation period. However, it remains unknown when and how Case 1 was exactly infected due to the lack of detailed epidemiological information with his close contactors.

All three cases were fully vaccinated and had mild clinical symptoms after infection, indicating that the immune responses elicited by vaccines might have alleviated the disease progression course. Cases 2 and 3 only briefly lived together with Case 1 in the same building for two days. This further underlines how contagious the new Omicron variant is. However, Case 2’s granddaughter also lived together with Cases 2 and 3 but she remained negative for the infection up to date, suggesting some limiting factors for the transmissibility of the virus. Whether the Omicron variant presents any clinical differences in China will require more cases to study. It will also be important to understand how Cases 2 and 3 were infected while the granddaughter was not in the same environment. Taken together, the relatively long incubation time in Case 1 but short incubation time in Cases 2 and 3, mild symptoms, and the unique set of mutations will be important to follow up to better control the possible further spread of this new Omicron variant in China.

## Data Availability

The SARS-CoV-2 sequences reported in this paper have been deposited in the China National Microbiology Data Center (http://www.nmdc.cn/). The access numbers are NMDCN0000RC9 for case 1, NMDCN0000RC7 case 2 and NMDCN0000RC8 case 3.
